# Neural Mechanisms Responsible for Vagus Nerve Stimulation-Dependent Enhancement of Somatosensory Recovery

**DOI:** 10.21203/rs.3.rs-3873435/v1

**Published:** 2024-01-29

**Authors:** Kaitlyn M. Malley, Andrea D. Ruiz, Michael J. Darrow, Tanya Danaphongse, Stephanie Shiers, Fatima N. Ahmad, Clareth Mota Beltran, Benjamin T. Stanislav, Theodore Price, Robert L Rennaker II, Michael P Kilgard, Seth A Hays

## Abstract

Impairments in somatosensory function are a common and often debilitating consequence of neurological injury, with few effective interventions. Building on success in rehabilitation for motor dysfunction, the delivery of vagus nerve stimulation (VNS) combined with tactile rehabilitation has emerged as a potential approach to enhance recovery of somatosensation. In order to maximize the effectiveness of VNS therapy and promote translation to clinical implementation, we sought to optimize the stimulation paradigm and identify neural mechanisms that underlie VNS-dependent recovery. To do so, we characterized the effect of tactile rehabilitation combined with VNS across a range of stimulation intensities on recovery of somatosensory function in a rat model of chronic sensory loss in the forelimb. Consistent with previous studies in other applications, we find that moderate intensity VNS yields the most effective restoration of somatosensation, and both lower and higher VNS intensities fail to enhance recovery compared to rehabilitation without VNS. We next used the optimized intensity to evaluate the mechanisms that underlie recovery. We find that moderate intensity VNS enhances transcription of Arc, a canonical mediator of synaptic plasticity, in the cortex, and that transcript levels were correlated with the degree of somatosensory recovery. Moreover, we observe that blocking plasticity by depleting acetylcholine in the cortex prevents the VNS-dependent enhancement of somatosensory recovery. Collectively, these findings identify neural mechanisms that subserve VNS-dependent somatosensation recovery and provide a basis for selecting optimal stimulation parameters in order to facilitate translation of this potential intervention.

## Introduction

Vagus nerve stimulation (VNS) combined with rehabilitative training has emerged as a novel strategy to improve recovery after neurological injury [1, 2]. This application of VNS therapy is founded on the principles of neurorehabilitation, which aims to facilitate recovery by promoting adaptive changes in neural circuits after injury. Brief bursts of electrical stimulation of the vagus nerve drive release of neuromodulators, including acetylcholine and norepinephrine, which are associated with synaptic plasticity [3–8]. Pairing VNS with forelimb motor training enhances synaptic connectivity changes in descending motor circuits and improves recovery across a range of preclinical models of neurological injury [9–17]. Moreover, based on a successful pivotal trial, VNS paired with upper limb rehabilitation recently received FDA approval for individuals with chronic stroke [18].

Building on these findings, a series of recent studies show that a congruent approach of pairing VNS with tactile rehabilitation promotes recovery of somatosensation. VNS combined with passive mechanical stimulation of hyposensitive skin surfaces reduces detection thresholds in a rat model of profound sensory loss, indicating a restoration of an aspect of somatosensory function [19–22]. Highlighting the clinical potential of this approach, a case study in an individual with profound sensory loss resulting from stroke provides initial evidence that pairing VNS with tactile retraining may improve recovery of somatosensation [23].

The ability to apply VNS to improve recovery of both motor and sensory function raises two overlapping questions, one theoretical and one practical. First, does VNS engage a similar underlying mechanism to enhance recovery of somatosensory function? VNS-directed recovery is known to require synaptic plasticity specific to the cortical networks engaged by the rehabilitative paradigm [16, 17]. For instance, pairing VNS with motor training yields refinement of synaptic connectivity in corticospinal networks controlling the trained muscles [17]. Moreover, related studies show that VNS-dependent plasticity and recovery follows a well-described inverted-U relationship with stimulation intensity [24–28]. Consequently, stimulation intensity provides a simple, accessible means to probe whether a similar mechanism, namely, enhancement of plasticity, underlies recovery of somatosensory function.

Second, what is the best strategy to apply VNS to improve recovery? Optimization is a key step in effective translation of VNS therapy. Emerging evidence provides a basis to define the optimal strategies for the accompanying tactile rehabilitation [20–22], but no studies directly examine the electrical stimulation parameters that yield the greatest recovery. Stimulation intensity is a critical determinant of VNS-dependent effects, thus optimizing intensity is a logical first step in developing this approach [28]. Both of these questions can be addressed by evaluating the effect of stimulation intensity on VNS-dependent recovery across a range of clinically-viable parameters known to predictably influence synaptic plasticity.

In our first experiment, we characterized the influence of VNS intensity on somatosensory recovery in a rat model of profound sensory loss. Mirroring results from pairing VNS with motor rehabilitation, we find that VNS exhibits an inverted-U relationship with stimulation intensity. Additionally, we provide two other lines of evidence indicating that pairing VNS with touch promotes synaptic plasticity. In our second and third experiments respectively, we found that VNS increases transcription of a plasticity-related gene in the cortex, and preventing plasticity abrogates VNS-dependent enhancement of somatosensory recovery. These findings help to define the mechanistic basis for VNS-dependent improvements in somatosensation and provide a basis for selecting optimal stimulation parameters.

## Methods

### Subjects

All methods followed the Animal Research: Reporting of In Vivo Experiments (ARRIVE) guidelines. 126 female Sprague-Dawley rats weighing approximately 250–300 g were included in this study (Charles River Labs). The rats were housed in a 12:12 reversed light cycle environment, and behavioral training was performed during the dark cycle to increase daytime activity levels. All handling, housing, stimulation, and surgical procedures were approved by the University of Texas at Dallas Institutional Animal Care and Use Committee (Protocols: 14 – 10 and 99 – 06) and conducted according to the protocols and guidelines.

## Peripheral Nerve Injury (PNI)

All animals underwent transection and repair of the median and ulnar nerves to generate chronic sensory loss, as described in previous studies [17, 29, 30]. Animals were deeply anesthetized with ketamine hydrochloride (50 mg/kg, IP), xylazine (20 mg/kg, IP), and acepromazine (5 mg/kg) and supplemented as necessary to maintain areflexia. The animal was placed in a supine position, and a sagittal incision was made 1cm proximal to the elbow of the right forelimb. The surrounding tissue was blunt dissected to expose, separate, and transect the median and ulnar nerves. The proximal and distal nerve stumps were sutured 1 mm from the ends of an 8 mm saline-filled polyurethane tube (Micro-Renathane 0.095” I.D 0.066” O.D., Braintree Scientific, Inc., Braintree, MA), resulting in a 6 mm gap between nerve stumps. The skin incision was sutured and covered in a triple antibiotic ointment. After surgery, animals were given subcutaneous injections of sustained release buprenorphine (10 mg/kg) and 10 ml of a solution of lactated ringers with 5% dextrose. Animals were outfitted with an Elizabethan collar for 4 days to prevent autophagia. Animals were allowed to recover for 8 weeks prior to the resumption of behavioral testing.

## Vagus Nerve Cuff Implantation

All animals were implanted with a stimulating cuff electrode on the left cervical vagus nerve, according to standard procedures [17, 20, 29]. Animals were anesthetized and placed in a stereotaxic apparatus to stabilize the skull. A midline incision was made to expose the surface of the skull. Four holes were drilled surrounding lambda, and bone screws were inserted to anchor a two-channel connector to the skull using acrylic. The animal was then removed from the stereotaxic apparatus and placed in a supine position. An incision was made 2 mm anterior and 2 mm lateral to the top of the sternum, and blunt dissection exposed the left cervical vagus nerve. After the nerve was isolated from the carotid artery, a stimulating cuff electrode was placed around the vagus nerve, and leads were tunneled subcutaneously to the head-mounted connector. VNS-dependent activation of the Hering-Breuer reflex was used to confirm correct placement and functionality of the stimulating cuff electrode. If five trains of stimulation (0.8 mA, 30 Hz, 100 microsecond pulse width, up to 5 train duration, with an inter-stimulus duration of 30 s) failed to decrease blood oxygen saturation, the cuff electrode was replaced [31]. All incisions were sutured closed, and the two-channel connector was enclosed in acrylic. After surgery, animals were given subcutaneous injections of buprenorphine (1.2 mg/kg) and 10ml of a solution of lactated ringers with 5% dextrose.

## Tactile Rehabilitation

8 weeks after PNI, 85 animals were allocated into one of 8 groups to receive tactile rehabilitation paired with VNS. 60 animals were included in the first experiment investigating the effect of current amplitude on recovery groups (No VNS (n = 15), Low VNS (n = 15), Moderate VNS (n = 15), High VNS (n = 15)). 15 animals were included in the second experiment, which examined the effect of VNS on plasticity-related gene expression (Arc No VNS (n = 7), Arc VNS (n = 8)). 10 animals were included in the third experiment to determine the necessity of plasticity for recovery (ACh+; VNS (n = 4), ACh-; No VNS (n = 6)).

Tactile rehabilitation began 9 weeks after PNI and was delivered similar to previous studies [19, 21, 22]. Animals received one session of rehabilitation daily, 4 days per week, for up to 5 weeks. Animals were placed in an acrylic booth with a mesh floor. The headmount was connected to an isolated pulse stimulator to permit delivery of VNS (Model 2100, A-M Systems, Sequim, WA, ± 100 V). Approximately every 10 seconds, a small paintbrush was applied to ventral surface of the right, previously denervated paw, to provide a mechanical stimulus. A short burst of VNS was triggered to coincide with the delivery of mechanical stimulation. Each stimulation consisted of a 500 ms train of 100 us biphasic pulses delivered at 30 Hz. VNS intensity was delivered as appropriate for each group: 0 mA (no VNS), 0.4 mA (low intensity), 0.8 mA (moderate intensity), or 1.2 mA (high intensity), with all other stimulation parameters held constant. All animals received 200 tactile VNS pairings per session, as in previous studies[20–22, 29].

## Mechanosensory withdrawal threshold testing

Mechanosensory withdrawal thresholds were assessed in all animals according to standard procedures [20, 29]. Testing was performed in an acrylic chamber (19.5 × 9.6cm) with a mesh wire floor. Animals were allowed to acclimate to the behavioral chamber for 30 minutes before testing commenced. Mechanical withdrawal thresholds of the left and right forepaws were tested using a dynamic plantar aesthesiometer (Cat. No. 37450; Ugo Basile, Lugano, Switzerland). The actuator filament (0.5 mm diameter) was positioned against the plantar surface of the forepaw, and a linearly increasing force was applied (20-second ramp time, 50 g maximal force). The force at which paw withdrawal occurred was captured for analysis. Testing alternated between the left and right forepaws, with a minimum of 1 minute between consecutive tests. Trials resulting in paw withdrawal due to spontaneous exploratory activity were excluded from analysis. Assessments were collected before injury, before rehab, and once weekly during rehab.

## Cylinder Asymmetry Testing

Spontaneous forelimb use was measured using the cylinder task as previously described [20, 29]. Animals were placed in a transparent Plexiglas cylinder (20 cm diameter) and allowed to freely explore for 3 minutes. Video was recorded from directly underneath the cylinder through a clear sheet of acrylic. The total number of left and right forepaw contacts with the wall during rearing was recorded. The relative use of the injured right forepaw was evaluated with an asymmetry index, calculated as (right/[left + right]) × 100. Assessments were collected before injury, before rehab, and once weekly during rehab by experimenters blinded to experimental conditions of the animals.

## Toe Spread Assessment

Toe spread analysis was performed to measure weight bearing using the stamp and paper method as previously described [20, 29]. The forepaws of the animals were coated in a layer of nontoxic ink, and the animals walked across a sheet of paper. The paper was scanned, digitized, and scored using ImageJ software (NIH, Bethesda, MD). Three footprints from both the left and right paw were analyzed by a blinded experimenter. The distance between the center of the second and fifth digits was measured and recorded for each print. Assessments were performed before injury, before rehab, and on the last week of rehab by experimenters blinded to the groups.

## mRNA Sequencing

One day after the last tactile rehabilitation session, rats were decapitated and the forelimb section of the contralateral somatosensory cortex was rapidly dissected in ice-cold dissection buffer and homogenized using Precellys Minlys Tissue Homogenizer (Bertin-instruments, P000673-MLYS0-A). Samples were homogenized at 10 second intervals (medium speed) for 10 minutes, at 4°C, in Precellys homogenizing CKMix tubes. RNA was performed on all samples using the Direct-zol RNA Microprep kit (R2060, Zymo Research) according to the manufacturer's instructions. RNA was eluted with 30 µL of RNase free water. RNA yield was quantified using a Nanodrop system (Thermo Fisher Scientific), and RNA quality was determined by a fragment analyzer (Advanced Analytical Technologies).

Total RNA from 15 cortical samples was purified using TRIzolTM (ThemoFisher) and subjected to Tru-seq stranded mRNA library preparation according to the manufacturer's instructions (Illumina). Tru-seq total RNA library kit with ribosomal RNA depletion was used to generate sequencing libraries. After standardizing the amount of cDNA per sample, the libraries were sequenced on an Illumina NextSeq500 sequencing platform with 75-bp single-end reads in multiplexed sequencing experiments, yielding a median of 22.3 million reads per sample. mRNA library preparation and sequencing were done at the Genome Center in The University of Texas at Dallas Research Core Facilities.

## Cortical cholinergic depletion

Cholinergic lesions were performed at the time of vagus nerve cuff implantations, similar to previous reports [4, 32–34]. Rats were anesthetized with ketamine hydrochloride (80 mg/kg, i.p.) and xylazine (10 mg/kg, i.p.), and given supplemental doses as needed to maintain anesthesia levels. After placing the rat in a stereotaxic frame (David Kopf Instruments, Tujunga, CA) burr holes were drilled bilaterally over the nucleus basalis. Rats received injections of either conjugated 192-IgG-saporin (Advanced Targeting Systems, San Diego, CA) to selectively lesion cholinergic neurons in the basal forebrain, or vehicle injections of an untargeted antibody and saporin, which does not enter cells and induce cell death. 192-IgG-saporin or vehicle (0.375 mg/mL in saline) were injected through a pulled glass needle at 0.1 µL/min using a Nanoliter 2010 injector (World Precision Instruments, Sarasota, FL). Injections were made at the following sites (site 1&2: 0.3 µL, AP: -1.4, ML: ±2.5, DV: -8.0; sites 3&4: 0.2 µL, AP: -2.6, ML: ±, DV: -7.0 mm). The needle remained in place for 4–5 minutes after each injection to allow for diffusion and prevent backflow. Burr holes were sealed with bone wax.

## Exclusion Criteria

41 animals were excluded from the study. Animals were excluded for the following reasons, which were pre-defined and consistent with our prior studies: pre-injury withdrawal threshold was above 15g (n = 7), post-injury withdrawal threshold was below 15g (n = 15), failure of the stimulating cuff (n = 5), failure of the headmount (n = 4), autophagia (n = 2), or mortality prior to completion of the full course of rehabilitation (n = 8). After exclusions, 85 rats were included in 1 of 8 groups (‘No VNS’ (n = 15), ‘Low VNS’ (n = 15), ‘Moderate VNS’ (n = 15), ‘High VNS’ (n = 15), ‘Arc No VNS’ (n = 7), ‘Arc VNS’ (n = 8), ‘ACh+; VNS’ (n = 4), ‘ACh-; VNS’ (n = 6)).

## Statistics

The data was first tested for normality using the Kolmogorov-Smirnov test (KS test). Upon testing distributional variance using the Kolmogorov-Smirnov test, we found that the mean and variance of our data were the same as a normal distribution, so we used parametric statistical comparisons. Mechanical withdrawal thresholds were analyzed using a two-way repeated measures ANOVA, followed by post hoc Bonferroni-Corrected unpaired t-test where appropriate. Paired t-test were used to compare measurements within subjects from pre-injury to week eight post-injury time points, where applicable. An unpaired t-test was used to analyze Arc transcript levels. A Pearson’s correlation coefficient was used evaluate association of Arc levels and magnitude of recovery. In the figures, data are presented as mean ± standard error of the mean (SEM).

## Results

We first sought to determine whether pairing VNS with tactile rehabilitation would improve somatosensory function in a current intensity-dependent manner. To do so, all rats underwent transection and repair of their median and ulnar nerves to induce chronic hyposensitivity in the ventral forepaw. As expected, withdrawal thresholds of the injured forepaw were significantly elevated 8 weeks after nerve injury (Before Injury v. After Injury; Paired t-test, t(58) = 1.67, p = 1.42 x $10^{-25}$).

Animals were then assigned to groups and underwent 4 weeks of daily tactile rehabilitation with VNS delivered at a fixed low, moderate, or high intensity or no stimulation, as appropriate for their group. Group analysis of withdrawal threshold recovery revealed a significant effect of group (1-way repeated measures ANOVA, No VNS v. Low VNS v. Moderate VNS v. High VNS; F[4, 228] = 7.75, p = 7.3 x $10^{-3}$. No differences in withdrawal thresholds were observed across groups prior to beginning rehabilitation (Fig. 1A; Unpaired t-tests, No VNS v. Low VNS v. Moderate VNS v. High VNS; t(30) = 1.70, p = 0.84, t(30) = 1.70, p = 0.71, t(30) = 1.70, p = 0.99).Consistent with previous reports, Moderate VNS paired with tactile rehabilitation significantly decreased withdrawal thresholds compared to equivalent tactile rehabilitation without VNS (Fig. 1A; Unpaired t-test, No VNS After Therapy v. Moderate VNS After Therapy, t(28) = 1.70, p = 3.2 x $10^{-3}$). These findings confirm that pairing moderate intensity VNS with tactile rehabilitation improves recovery of somatosensory function.

We next tested if VNS delivered at lower or higher intensities would similarly improve recovery. Low intensity VNS delivered at 0.4 mA failed to improve recovery compared to no stimulation (Fig. 1A; Unpaired t-test, No VNS After Therapy v. Low VNS After Therapy, t(28) = 1.70, p = 0.33). Similarly, High VNS did not improve recovery compared to no stimulation (Fig. 2A; Unpaired t-test, No VNS After Therapy v. High VNS After Therapy, t(29) = 1.70, p = 0.12). At the end of therapy, the Moderate VNS group was the only group to demonstrate a significant percent recovery over No VNS (Fig. 1B; One-way ANOVA, F[3, 55],

= 4.45, $p = 7.2 \times 10^{-3}$; Unpaired t-tests, Percent Recovery No VNS v. Low VNS, v. Moderate VNS, and High VNS; t(27) = 1.70, p = 0.12; t(28) = 1.70, $p = 8.7 \times 10^{-3}$; t(28) = 1.70, p = 0.14). Additionally, when we stratified animals by the magnitude of somatosensory recovery, a chi-squared test of independence revealed that moderate intensity VNS resulted in a greater proportion of fully recovered animals (as defined by a > 90% restoration of pre-injury withdrawal threshold), with 93% of animals demonstrating full recovery compared to the No VNS group where only 7% of animals completely recovered (Fig. 1C; $X^2$ Test for Independence, Degree of Recovery: No VNS v. Moderate VNS; $X^2$ (2, N = 30) = 12.1, $p = 2.3 \times 10^{-3}$). Low and high intensity VNS resulted in approximately 65% of animals with full recovery but the proportion of animals who made a complete recovery did not statistically differ from the No VNS group (Fig. 1C; $X^2$ Test for Independence, Degree of Recovery: No VNS v. Low VNS and High VNS; $X^2$ (2, N = 29) = 3.06, p = 0.22; $X^2$ (2, N = 30) = 3.45, p = 0.18). These findings define an inverted-U relationship between stimulation intensity and recovery of somatosensory function.

We next tested if VNS-dependent improvements in somatosensory function would generalize to other measures of forelimb sensorimotor function. No improvements in forelimb use during spontaneous exploration or weight bearing were observed across groups (Fig. 2A, 1-way repeated measures ANOVA, F[1,49] = 0.12, p = 0.73; Fig. 2B, 1-way repeated measures ANOVA, F[1,51] = $2.1\mathrm{X}10^{-4}$, p = 0.99). These findings suggest that VNS benefits do not generalize.

The finding that VNS-dependent recovery exhibits an inverted-U relationship with stimulation intensity provides circumstantial evidence that plasticity may underlie recovery. In our second and third experiments, we sought to probe this mechanism more directly. Prior studies show that VNS paired with other forms of training modulates expression of proteins associated with synaptic plasticity, including Activity-regulated cytoskeleton-associated protein (Arc) [35–37]. Our second experiment assessed whether VNS paired with tactile rehabilitation increased the expression of Arc transcript in cortex compared to tactile rehabilitation without VNS in a separate cohort of animals. Because moderate intensity VNS produced the largest improvement in recovery, we utilized this parameter set in the subsequent experiments. A separate cohort of animals underwent one week of tactile therapy with either moderate intensity VNS (n = 8) or no stimulation (n = 7). VNS paired tactile rehabilitation significantly increased Arc transcript levels in cortex compared to equivalent tactile training with no stimulation (Fig. 3A; Unpaired t-test, Arc No VNS v. Arc VNS; t(13) = 2.62, p = 0.021). Additionally, Arc transcript levels were weakly, but significantly, correlated with the degree of recovery (Fig. 3B; Pearson correlation, $r^2$ = 0.27, p = 0.0492). These observations are consistent with a scenario in which VNS-directed plasticity enables recovery.

If indeed cortical plasticity underlies VNS-dependent recovery of somatosensory function after nerve injury, then manipulations that prevent this plasticity should block recovery. In our third experiment, we directly explored this in a separate cohort of animals using IgG-192-saporin to deplete forebrain acetylcholine, a common technique known to abrogate plasticity [4, 38]. Animals underwent nerve injury as in the initial experiment and were randomized to receive either IgG-192-SAP injections to deplete acetylcholine (ACh-; VNS, n = 6) or vehicle injections (ACh+; VNS, n = 4). All animals received equivalent moderate intensity VNS paired with tactile rehabilitation. ANOVA revealed a significant effect of treatment (1-way repeated measures ANOVA, VNS; ACh + v. VNS; ACh-, $F[6,48] = 4.19$; $p = 2.9 \times 10^{-5}$). After VNS therapy, animals with depletion of acetylcholine demonstrated significantly worse recovery compared to animals with intact cholinergic signaling (Fig. 4; Unpaired t-test, After Therapy; ACh+; VNS v. ACh-; VNS, $t(8) = 9.93$, $p = 8.93 \times 10^{-6}$). These findings are consistent with the hypothesis that VNS enhances somatosensory recovery by directing synaptic plasticity.

## Discussion

In our first experiment, we characterized the effects of stimulation intensity on VNS-dependent enhancement of somatosensory recovery in a rat model of chronic sensory loss. Consistent with prior studies, we find that VNS, delivered at a moderate stimulation intensity, combined with tactile rehabilitation enhances recovery of forelimb somatosensory function. Higher and lower stimulation intensities fail to produce comparable improvements in recovery. The inverted-U relationship between stimulation intensity and recovery suggests that VNS-dependent enhancement of plasticity may underlie recovery. Consistent with this, we next show that VNS paired with tactile training enhances the levels of Arc transcript in somatosensory cortex, and that preventing plasticity blocks VNS-dependent recovery. Together, findings from these experiments reveal mechanisms that underlie VNS-dependent improvements in somatosensation and provide guidance to select optimal stimulation parameters for future use.

The core finding of this study confirms that moderate intensity VNS paired with tactile rehabilitation enhances recovery of somatosensation. These findings corroborate previous studies demonstrating VNS-dependent somatosensory improvements in both preclinical models and a pilot study in an individual with stroke, all of which utilize moderate intensity stimulation [19–23]. Additionally, we observe that lower and higher stimulation intensities fail to generate significant improvements in somatosensory recovery compared to tactile rehabilitation without stimulation. The findings provide a straightforward basis for selection of stimulation intensity for future use of this approach. Combined with prior work characterizing the parameters regarding timing and dose of VNS therapy [21, 22], as well as the influence of the paired tactile stimuli [20], this study adds a critical dimension to optimizing VNS therapy for clinical implementation.

The inverted-U relationship of VNS intensity has been reported across a broad range of contexts, from memory enhancement to extinction learning to motor recovery [28, 39–41]. A number of explanations could account for this phenomenon, but two likely schema, which have been described extensively in prior literature, involve either desensitizing or functionally opposing action of neuromodulatory networks [39]. Regardless of the specific molecular pathways, the pervasive occurrence of this phenomenon implies that VNS likely engages a common, central mechanism. Synaptic plasticity is a common denominator linking prior studies of VNS, such that VNS exhibits an inverted-U relationship with cortical plasticity [25–27], and the effective parameters mirror those for VNS-dependent recovery of forelimb function [28]. We hypothesized that a similar enhancement of plasticity, when combined with tactile rehabilitation, would underlie recovery of somatosensory function. The observation of the inverted-U relationship reported here provides initial, though indirect, evidence of this mechanism.

This claim is bolstered by two experiments that more directly examine VNS-dependent plasticity. First, we observed that VNS paired with tactile rehabilitation enhances expression of Arc transcript in the somatosensory cortex compared to tactile rehabilitation without VNS. Arc is an immediate early gene implicated in many canonical forms of synaptic plasticity [37, 42]. The increase in Arc observed here is consistent with prior studies that report changes in Arc mRNA and protein levels with VNS delivery [35, 36]. Moreover, the correlation of Arc mRNA levels and VNS-dependent enhancement of somatosensory recovery suggests that plasticity underlies recovery. If so, then manipulations that prevent plasticity should consequently block VNS-dependent recovery. We explored with hypothesis in a third experiment using targeted immunodepletion of acetylcholine, a technique known to prevent VNS-dependent plasticity [17]. We find that acetylcholine depletion blocks VNS-dependent enhancement of somatosensory recovery. Taken together, these three lines of evidence, the inverted-U relationship with intensity, the VNS-driven increase in Arc levels, and the abrogation of recovery when plasticity is limited, strongly indicate that VNS-dependent plasticity underlies recovery of somatosensory function.

VNS therapy recently received FDA approval to improve recovery of upper limb arm and hand function in individuals with chronic stroke, and a number of other applications of this approach are rapidly emerging. As VNS therapy is increasingly developed and translated to clinical use, a clear understanding of the mechanisms that underlie recovery is important for optimization. The findings from this study clarify the mechanisms engaged by VNS therapy to promote recovery of somatosensory function and provide clear guidelines for selecting effective stimulation parameters for future studies.

## Declarations

### Data Availability

The datasets generated during and/or analysed during the current study are available from the corresponding author on reasonable request.

### Author Contributions

K.M.M.: Study design, surgical procedures, behavioral tests, supervision of data collection, data analysis, preparation of the figures and manuscript. A.D.R.: Study design, surgical procedures, behavioral tests, supervision of data collection. M.J.D.: Study design, behavioral tests, supervision of data collection, data analysis, preparation of the figures. T.T.D.: Surgical procedures. S.S.: Supervision of data collection, data analysis. F.N.A. and C.M.B.: Behavioral tests. B.T.S.: Behavioral tests, data analysis. T.P.: Supervision. R.L.R.: Supervision. S.A.H.: Conceptualization, study design, supervision, data analysis, and manuscript revisions. M.P.K.: Project administration, conceptualization, study design, data analysis, and manuscript revisions.

#### Acknowledgements

We would like to thank Alfonso Reyes for the construction of VNS stimulator cuffs. We also thank Megan White, Joseph Montefalcon, Ronse Raphi, Mamoon Aslam-Syed Mian, Nikit Valmiki, Immaculate Wambugu, Ayesha Yousaf, Sakina Husain, Afaf Nabeeha, and Abhishek Sule for their help in behavioral training and testing. Finally, thank you to David Pruitt his technical expertise and guidance throughout the experimental process.

#### Sources of Funding

This work was supported by the National Institutes of Health NIH R01 NS094384 (SAH) and by the Department of Defense Joint Program Committee 8/Clinical and Rehabilitative Medicine Research Program and the Congressionally Directed Medical Research Programs grant W81XWH-20-1-0863 (SAH).

#### Conflict of Interest Statement

MPK has a financial interest in MicroTransponder, Inc. RLR is the co-founder and CEO of XNerve, which is developing nerve stimulation therapies. The other authors declare that no competing interests exist.

## Figures

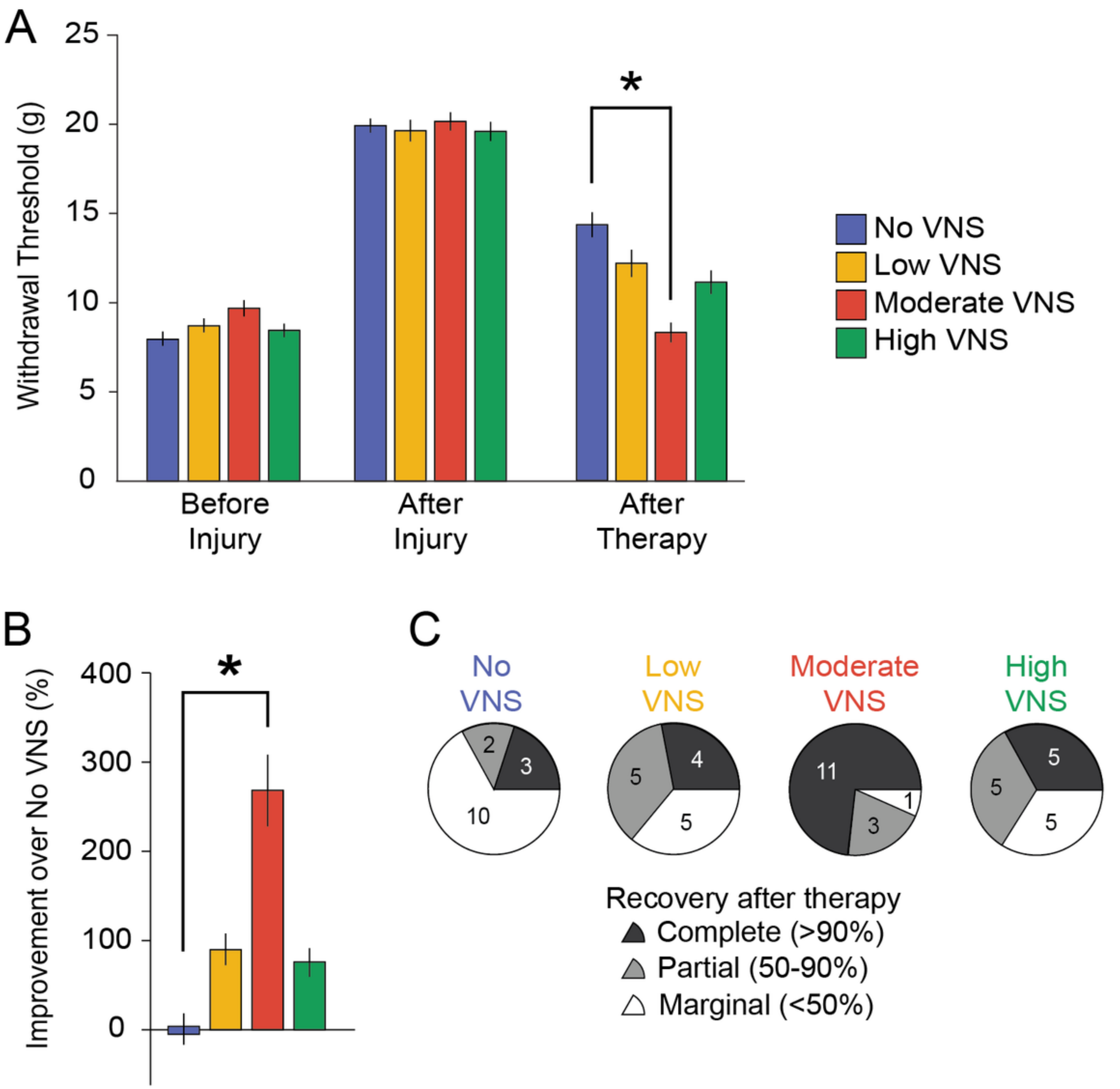

**Figure 1**

**Moderate VNS improves recovery of somatosensory thresholds**. **(A)** Nerve injury worsens withdrawal thresholds in all groups, indicative of chronic hyposensation. Moderate VNS paired with tactile rehabilitation enhances recovery compared to no stimulation with equivalent tactile rehabilitation after therapy. Low and high intensity VNS fail to produce equivalent improvements in recovery. **(B)** Only moderate intensity VNS produces significant improvements in recovery. **(C)** Moderate intensity VNS results in the greatest proportion of animals exhibiting full recovery of withdrawal thresholds after therapy. * indicates $p < 0.05$.

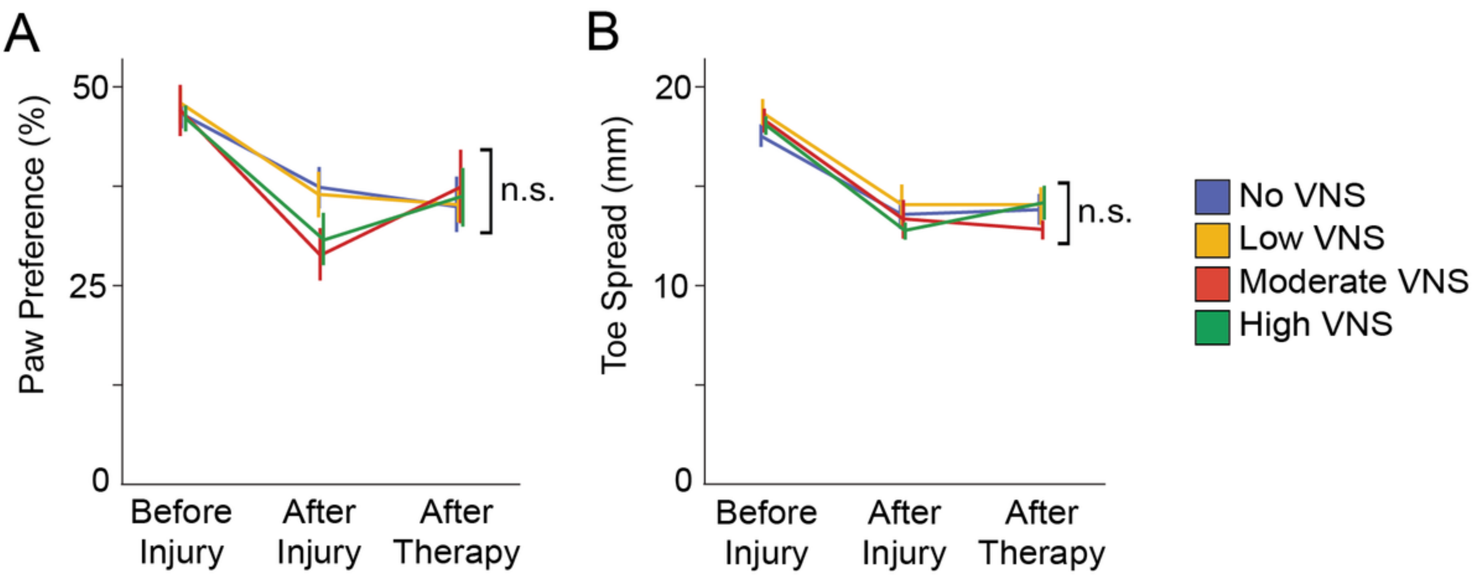

**Figure 2**

**VNS-dependent recovery does not generalize to other measures of forelimb function.** None of the tested VNS paradigms improved recovery of **(A)** volitional forelimb use during exploratory behavior or **(B)** weight bearing during locomotion. n.s. denotes no significant difference.

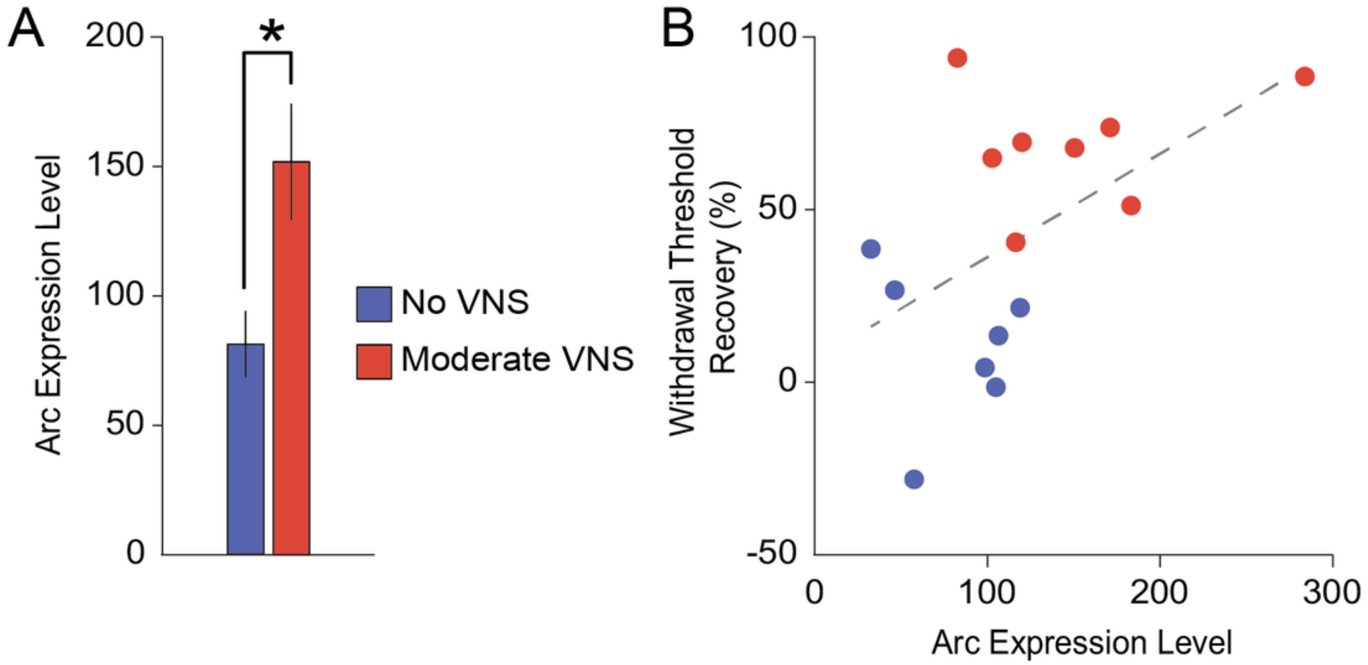

**Figure 3**

**VNS increases Arc mRNA transcript levels**

(A) VNS paired with tactile rehabilitation (n = 8) significantly increases the levels of Arc mRNA in cortex compared to equivalent tactile rehabilitation without VNS (n = 7). (B) Arc mRNA levels are weakly, but significantly, correlated with the degree of forelimb somatosensory recovery. * indicates $p < 0.05$. Data are presented as mean ± SEM in panel A, and panel B shows datapoints from individual subjects.

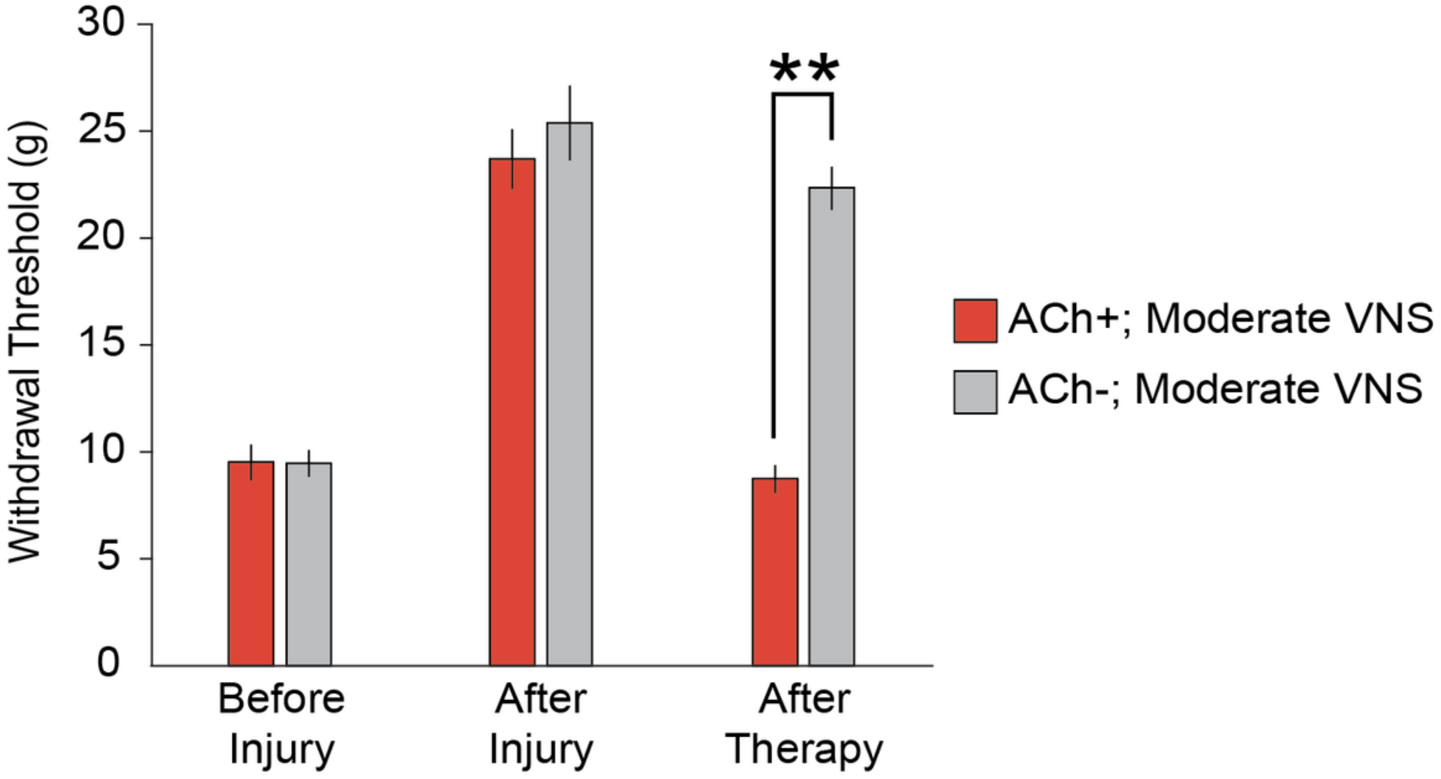

**Figure 4**

**Blocking plasticity prevents VNS-dependent enhancement of somatosensory recovery.**

VNS paired with tactile rehabilitation significantly improves recovery of forelimb somatosensation in control rats (ACh+; VNS, n = 4). Depletion of forebrain acetylcholine, which blocks cortical plasticity, significantly impairs VNS-dependent recovery (ACh-; VNS, n = 6). ** indicates $p < 0.001$ across groups. Data are presented as mean ± SEM.
